# Additive Manufacturing in Organic Chemistry: From Synthesis to Sustainable Process Design

**DOI:** 10.3390/ijms27083512

**Published:** 2026-04-14

**Authors:** Adrian Domiński, Barbara Zawidlak-Węgrzyńska, Joanna Rydz

**Affiliations:** 1 Centre of Polymer and Carbon Materials, Polish Academy of Sciences, 41-819 Zabrze, Poland; adrian.dominski@gmail.com; 2 Department of Chemistry, Faculty of Medicine in Zabrze, Academy of Silesia, 40-555 Katowice, Poland; barbara.zawidlak@wst.com.pl

**Keywords:** additive manufacturing, 3D printing, polymer, organic synthesis, laboratory devices

## Abstract

Additive manufacturing (AM) is a process that creates a three-dimensional (3D) physical object from a digital design by building layers of material directly from a computer-aided design (CAD) file, allowing for precise and rapid production of parts or prototypes. AM is increasingly recognised as a sustainable production method due to its potential to reduce waste, energy consumption, and environmental impact. The versatility and efficiency of AM have made it an essential tool for rapid prototyping and developing custom parts and components with intricate designs that were previously difficult or impossible to produce. This review highlights the significant progress in utilising AM for the synthesis of organic compounds and the fabrication of organic devices. AM technologies are used in the synthesis of organic compounds, particularly through the use of 3D-printed catalysts, reactors and flow systems. Advances in AM have enabled this technology to be used to synthesise organic compounds and produce low-cost, customised organic equipment. This makes it possible to obtain sophisticated reactors, laboratory equipment or their individual parts, tailored to a specific chemical process in more sustainable way. AM has great potential for advancing green and sustainable chemical processes, with the ability to integrate multiple enabling technologies and facilitate safer and more efficient processes in a cost-effective manner. Overall, the integration of AM in organic synthesis has opened up new possibilities for innovative solutions in the field.

## 1. Introduction

Additive manufacturing (AM), also in a non-technical context, three-dimensional (3D) printing, revolutionises traditional manufacturing processes by allowing for the creation of complex and customised objects directly from digital designs using computer-aided design (CAD) [1]. AM involves layer-by-layer fabrication, enabling the creation of complex geometries and topologies that are difficult or impossible to achieve using conventional synthesis methods [2]. This technology has the potential to significantly reduce time and cost in prototyping and production, and has applications across a wide range of industries, from aerospace and automotive to healthcare and consumer goods. With the ability to produce intricate geometries and lightweight structures, AM presents new opportunities for innovation and efficiency in manufacturing [3].

**Figure 1:**
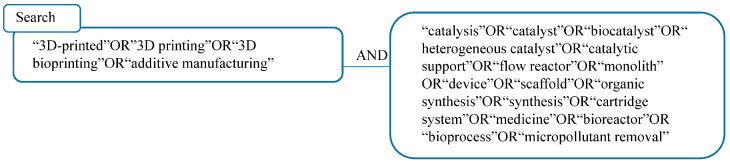
The main search keywords.

The scientific enthusiasm behind AM arises from the ability to fabricate low cost and customised devices with tailored solubility, stability, and reactivity, which facilitate research in the field of organic synthesis. AM technology has transformed the capabilities of chemists by allowing them to create customised devices, reactors, and lab hardware that may not be commercially available. This innovation enables researchers to push the boundaries of their work and develop more advanced solutions for their experiments. The ability to design and produce unique tools tailored to specific research needs has significantly enhanced the efficiency and precision of chemical studies [4]. The advances in the field of AM have meant that a properly selected technology can be used to make custom prototypes with complex geometry that can be made very precisely from various materials, including metals, ceramics, polymers, (bio)degradable materials, stainless, electro- or thermo-conductive, and heat- and chemo-resistant materials. Additionally, AM enables the fabrication of molecules, which can be used to create novel biological and pharmaceutical compounds, such as proteins, peptides, and small molecules. Such developments represent a significant leap in manufacturing capabilities and open up new possibilities for innovation in various industries [5,6,7,8]. Thus, giving opportunities to obtain sophisticated reactors, lab apparatus or individual parts adapted to a specific process. In the field of organic synthesis, the most prominent studies are focused on to design and use of AM in catalysis and flow devices. This innovation allows for the design and customisation of equipment, ultimately enhancing efficiency and precision in scientific research and experimentation. AM has evolved beyond prototyping to produce complex, high-value finished goods, enabling customisation and individualisation in organic synthesis through the creation of intricate reactors and catalysts. With its benefits including design flexibility, reduced waste, improved biocompatibility across multiple biological hosts, rapid prototyping, and cost-effectiveness, AM holds great promise for further advancements in printing speed, resolution, and scalability, making it a valuable technology for various industries [9]. The research questions addressed in this article refer to the latest developments and cutting-edge innovations in a niche topic, the use of 3D printing in organic synthesis, and what possibilities and ideas for the development or improvement of printing technology are possible in order to identify important environmentally friendly solutions in AM technology that enable production in an environmentally friendly and cost-effective way. The main search keywords adopted are shown in Figure 1.

## 2. Comparison of Additive Manufacturing Technologies for Organic Synthesis and Processing

AM encompasses a diverse set of fabrication technologies that differ significantly in their operating principles, material compatibility, and achievable structural resolution. In the context of organic chemistry, the selection of an appropriate AM technology is crucial, as it directly influences the feasibility of reactor fabrication, chemical resistance, and the ability to perform controlled synthesis under static or flow conditions.

Among the most widely used AM technologies is material extrusion (MEX) technology (commercialised under the trademarked name of fused deposition modelling (FDM) and sometimes also commonly referred to as fused filament fabrication (FFF)). This method is based on the layer-by-layer deposition of thermoplastic filaments such as polylactide (PLA), acrylonitrile–butadiene–styrene terpolymer (ABS), or poly(ethylene glycol-*co*-1,4-cyclohexanedimethanol terephthalate) (PETG). Its main advantages include low cost, ease of operation, and accessibility. Consequently, MEX has been extensively employed for the rapid prototyping of reaction vessels, microfluidic devices, and auxiliary laboratory equipment. However, its relatively low resolution and limited chemical resistance, particularly toward organic solvents, restrict its applicability in more demanding synthetic environments [10].

Vat photopolymerisation (VPP) technologies, (formerly stereolithography (SL) or digital light processing (DLP)) offer significantly higher resolution and superior surface finish compared to MEX. These methods rely on the photopolymerisation of liquid resins, enabling the fabrication of intricate geometries with fine internal channels. Such features are particularly advantageous in the design of microreactors for flow chemistry applications, where precise control over mixing and residence time is required. Nevertheless, the chemical stability of photopolymer resins remains a critical limitation, as many commercially available materials exhibit poor resistance to aggressive organic solvents and elevated temperatures [10].

Powder bed fusion (PBF) technologies, such as selective laser sintering (SLS), provide an alternative approach by fusing polymer powders (e.g., nylon or polyamide) using a laser source. This method enables the fabrication of mechanically robust and chemically more resistant structures without the need for support materials. As a result, SLS is particularly suitable for producing complex reactor architectures and components intended for long-term use. Despite these advantages, the relatively high cost of equipment and limited availability of chemically inert materials constrain its widespread adoption in routine laboratory practice [10].

Direct metal laser sintering (DMLS) is PBF technology specifically used for the fabrication of metallic structures. In this technology, a high-power laser selectively sinters metal powder particles layer-by-layer to produce dense, mechanically robust components. Commonly used materials include stainless steels, titanium alloys, and aluminium-based alloys, which exhibit excellent thermal conductivity, mechanical strength, and chemical resistance. These properties make DMLS particularly attractive for applications in organic synthesis that require operation under harsh conditions, such as elevated temperatures and pressures [11]. In comparison to polymer-based AM technologies, DMLS offers superior durability and heat transfer characteristics, which are critical for process intensification and highly exothermic or endothermic reactions. For instance, metallic monoliths and structured reactors fabricated via DMLS have been successfully applied as catalyst supports in processes such as methane dry reforming, where efficient heat management and structural stability are essential. Moreover, the inherent metallic nature of the printed structures may contribute to catalytic activity, either directly or through subsequent surface functionalisation. However, despite these advantages, DMLS is associated with several limitations. The technology typically requires expensive equipment and controlled processing environments, which may limit its accessibility for routine laboratory use. In addition, the range of available materials, although expanding, is still more limited compared to conventional metallurgy. Surface roughness and post-processing requirements may also influence the final performance of the printed components, particularly in microfluidic applications. Overall, DMLS complements other AM technologies by enabling the fabrication of robust, high-performance metallic reactors and catalyst supports, thereby expanding the applicability of AM in organic synthesis and chemical process engineering [12].

Material jetting (MJT) and inkjet-based printing technologies such as direct ink writing (DIW) represent another class of AM technologies, offering high resolution and the possibility of multi-material fabrication. These methods allow for the integration of different functional components within a single printed device, such as transparent windows, flexible seals, or catalytically active domains. In organic chemistry, this opens new opportunities for the fabrication of advanced microreactors and lab-on-a-chip systems. However, similar to VPP, the range of chemically resistant materials remains limited [13].

**Figure 2:**
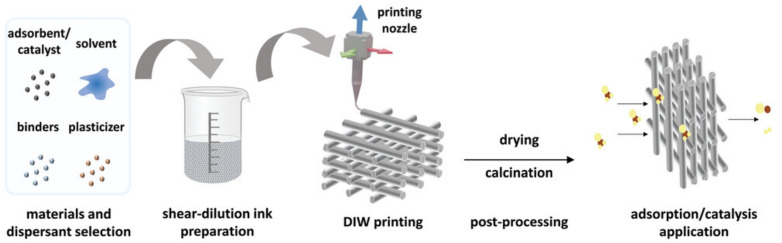
The complete preparation process of DIW for gas-phase adsorption and catalysis. After selecting a specific material, a shear-dilution ink is prepared by adjusting the proportions of solvent, binder, and plasticiser. Then the DIW fabrication is conducted and the prepared components are post-treated for further adsorption/catalysis application. Reprinted with permission from Ref. [14]. Copyright [2023] Wiley.

In the DIW process, printable pastes (inks) are formulated by incorporating suitable adsorbents or catalysts, with careful control of rheological properties to ensure printability and structural fidelity. Post-processing steps, such as drying and calcination, are typically employed to enhance the mechanical stability and functional performance of the resulting printed structures (Figure 2) [14].

To provide a clearer overview of the capabilities and limitations of different AM techniques, a systematic comparison is summarised in Table 1.

The integration of AM into organic chemistry requires a careful balance between technical capabilities and application-specific requirements. A systematic understanding of the advantages and limitations of each AM technology is essential for the rational design of 3D-printed systems, ranging from simple reaction vessels to complex, sustainable chemical processing platforms.

## 3. Organic Synthesis

The significance of catalysis in organic chemistry is fundamental. Humanity has been associated with catalysis, from ancient civilisations producing alcoholic beverages to the modern manufacturing of consumer goods and solutions to 21st-century environmental challenges. In this context, AM has emerged not merely as a fabrication tool, but as a platform for rational catalyst and reactor design, enabling precise control over geometry, porosity, and mass transport phenomena. Unlike conventional catalyst shaping methods, AM allows the decoupling of catalytic composition from structural architecture, which is particularly advantageous in heterogeneous catalysis.

**Figure 3:**
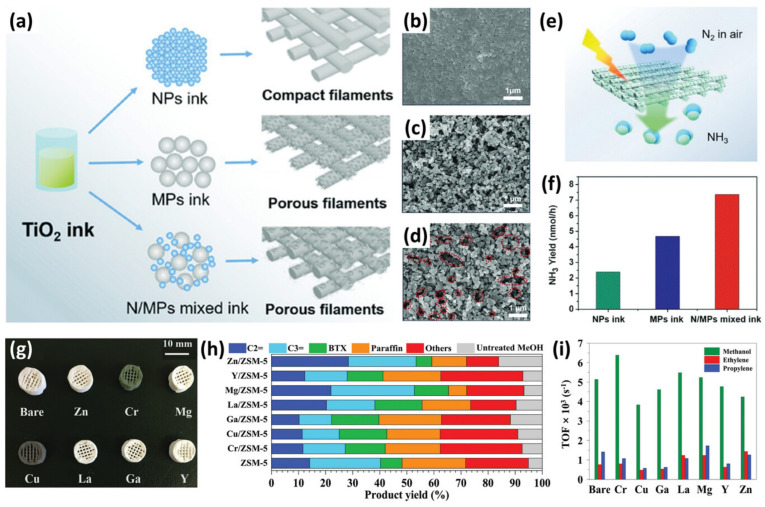
Catalytic reactor modification: (**a**) 3D printing of a titanium(IV) oxide (TiO_2_) scaffold using nanoparticles (NPs), submicron particles (MPs), and NPs/MPs mixed printing inks and their corresponding printed porous filaments. Scanning electron microscope (SEM) images of (**b**) NPs monolith, (**c**) MPs monolith, and (**d**) NPs/MPs monolith. Red ovals indicated NPs/MPs monolith had extra nano-/micro-pores for fast reactant diffusion inside the catalyst. (**e**) N_2_ fixation experiment conducted by printed TiO_2_ monoliths. (**f**) The catalytic reaction rates of the photocatalytic structure prepared by NPs, MPs, and NPs/MPs mixed printing inks, respectively. (**g**) ZSM-5 zeolite monoliths doped with various transition metals. (**h**) The products and their yields by catalysts doped with different metal ions. (**i**) The turnover frequency (TOF, representing the moles of methanol/product reacted or produced over per unit mole of acid sites per unit time) of metal-doped ZSM-5 in methanol-to-olefin reaction. Reprinted with permission from Ref. [14]. Copyright [2023] Wiley.

It is well-known that the greater the surface area-to-volume ratio of the catalyst, the more efficient the catalytic process occurs. The optimisation of catalysts to nano-sized levels is commonly used in academic studies, with high appliance potential in the chemical industry [26]. A key advantage of AM lies in the ability to engineer hierarchical porosity (macro-, meso-, micro-) and tailored-flow paths, which directly influence catalytic performance. Across the reported studies, a consistent trend emerges: enhanced catalytic efficiency is not solely determined by chemical composition, but is strongly governed by the geometrical and transport properties of the printed structure. Consequently, AM has evolved into a powerful platform for the shape engineering of porous materials and the fabrication of scalable catalytic systems for a wide range of applications. For example, in methanol-to-olefin (MTO) conversion, various 3D-printed metal-doped monoliths prepared via direct fabrication technology—robocasting (analogous to DIW)—have been systematically investigated (Figure 3i) [27]. By incorporating catalytic particles of multiple size scales, the voids between them naturally generate a hierarchical, interconnected pore structure that facilitates efficient gas transport within the catalytic component (Figure 3a–f).

The higher catalytic activity for methanol conversion to the light olefins was observed for the magnesium (Mg) and zinc (Zn)-doped ZSM-5 monolith (shape-selective catalyst—zeolite socony mobil-5 molecular sieves) (Figure 3g,h). The increased selectivity was attributed to the presence of moderate Bronsted acidity, which inhibited the formation of side products, i.e., aromatics and paraffins [28]. It was revealed that both acidity modulation and pore hierarchy are critical parameters controlling selectivity toward light olefins. It has also been demonstrated that AM technologies can be tailored for specific chemical reactions. In order to do this, 3D-printed hydrogen forms of ZSM-5 monoliths with a hierarchical pore network (macro-, meso-, microporous) incorporating amorphous silica into the monolith structure and surface-coated with silicoaluminophosphate (SAPO-34) zeolite were used for the methanol-to-olefin process. Importantly, these studies collectively highlight a broader principle that AM enables the transition from material-controlled catalysis to structure-controlled catalysis.

**Figure 4:**
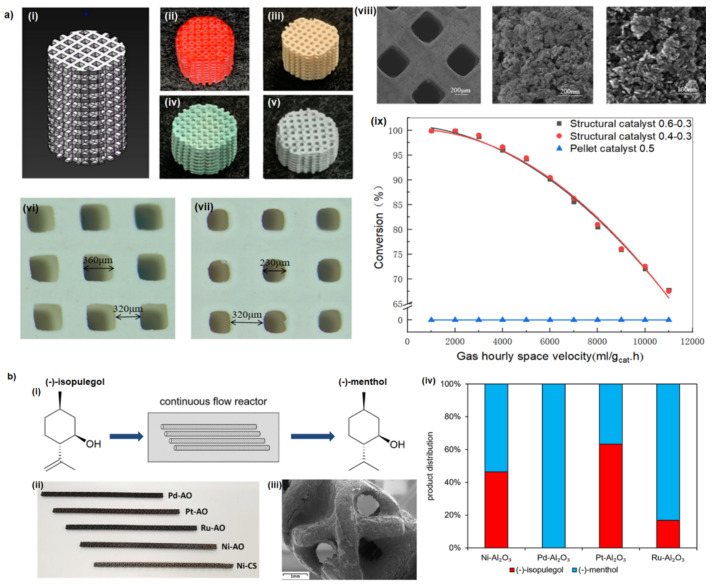
(**a**) Alumina catalyst fabrication steps: (i) modelling in SolidWorks, (ii) 3D-printed structure, (iii) carrier after sintering, (iv) carrier after impregnation, and (v) structured alumna catalyst. Catalyst carrier dimensions measured after printing for carriers: (vi) Al_2_O_3_-0.6-0.3 and (vii) Al_2_O_3_-0.4-0.3. (viii) SEM images of catalyst carriers at various magnifications. (ix) Plot of the conversion rate with space velocity at reaction temperature 300 °C; alumina catalyst carrier dimensions in the design model: 0.6 and 0.4 mm channel size and 0.3 mm spacing between channels. Reprinted with permission from Ref. [42]. Copyright [2021] American Chemical Society. (**b**) (i) Schematic process flow diagram of the reactor configuration used for continuous flow hydrogenation. (ii) Photography of the different reactor types used for this study. (iii) SEM image of palladium-supported catalyst (Pd–Al_2_O_3_). (iv) Column charts of the product distribution for the hydrogenation of (−)-isopulegol in *n*-heptane over four sets at 24 bar, 130 °C, H/S = 1.5, and VL = 1 mL/min. Reprinted with permission from Ref. [43]. Copyright [2021] American Chemical Society.

The application of 3D-printed catalysts compared to powdered catalysts demonstrated the improved selectivity of methanol conversion to light olefins [29]. The architecture dependence of the ZSM-5 catalyst was also reported for the methanol-to-olefin process. The catalyst featuring zig-zag channels in the direction of the flow demonstrated a higher selectivity to C2–C4 olefins compared to the monolith with straight channels [30]. In another study, 3D-printed zeolite-based monoliths were used as the heterogeneous catalyst for *n*-hexane cracking reactions. It was found that the 3D-printed system possessed higher selectivity to light olefins than its powder counterpart [31]. The 3D-printed zeolite heterogeneous catalysts were further used for the conversion of methanol [32,33] or CO_2_ [34] to dimethyl ether or the oxidative propane dehydrogenation process [35,36]. Another interesting example is using the high-resolution DLP technology to obtain a monolith with a controlled micron-scale porosity followed by deposition of zeolite layer. In this case, was demonstrated that cobalt tetraoxide (Co_3_O_4_)-spinel catalysts supported on monoliths obtained by DLP process were found to be catalytically active in the combustion of toluene [37]. A similar approach deals with the gas-phase isomerisation of monoterpene hydrocarbons. The corundum (aluminium oxide, Al_2_O_3_)–silicate monoliths were functionalised by deposition of zeolite layers with various atomic ratios of silicon/aluminium (Si/Al). By adjusting the Si/Al ratio the catalytic performance of gas-phase isomerisation of α-pinene can be controlled [38]. Very recently, PBF-printed metal lattices functionalised using zeolites were found to be useful in catalytic cracking of endothermic fuels for flight vehicles [39]. The Al-based Lewis acids are broadly used in catalytic applications, both as catalysts and as catalytic supports [40]. Therefore, a series of 3D-printed Al_2_O_3_-based catalysts with a broad range of catalytic applications were reported. The Al_2_O_3_ monolith with a woodpile porous structure obtained by robotic deposition followed by sintering at high temperature has been demonstrated as an effective and reusable heterogeneous catalyst in Biginelli and Hantzsch reactions. In this case, the high surface-to-volume ratio provides remarkable efficacy as a Lewis acid during reactions with higher yields up to 20% compared to the powder Al_2_O_3_ [41]. Similarly, a cubic γ-Al_2_O_3_ structured catalyst for hydrogen production from methanol process has been prepared via DLP technology (Figure 4a) [42].

The incorporation of catalytically active metals into 3D-printed Al_2_O_3_ monolith is common practice. An elegant example is the incorporation of metal catalysts (copper (Cu), palladium (Pd), platinum (Pt), ruthenium (Ru), and nickel (Ni)) into an alumina monolith, which has been studied for hydrogenation of flavourings and fragrances. The studies revealed that hydrogenation of (−)-isopulegol at mild conditions produced a commercially high-value product (−)-menthol with excellent yields and purity (Figure 4b) [43]. To date, the addition of catalyst metals into 3D-printed Al_2_O_3_ monolith was reported as a catalyst in a few types of reaction including Ullmann coupling (Cu/Al_2_O_3_) [44], cross-coupling reactions (Pd/Al_2_O_3_) [45], oxidative coupling of methane (manganese-sodium tungstate/aluminium oxide, Mn–Na_2_WO_4_/Al_2_O_3_) [46], carbon dioxide (CO_2_) methanation (Ni/Al_2_O_3_ [47,48] or Cu/Al_2_O_3_ [49]), and oxidation of benzyl alcohol into benzaldehyde (iron–cobalt (Fe–Co) alloy, Fe–Co/Al_2_O_3_ and Fe–Pd/Al_2_O_3_ [50]).

The same concept is often used for graphene oxide as a monolith for 3D-printed catalysts. Both Al_2_O_3_ and graphene usually share the same goal—to increase the surface-to-volume ratio. However, despite this commonality, graphene oxide gives additional advantages including electrical, thermal conductivity or better mechanical applications. The applications of graphene oxide in catalysis via AM, along with its advantages and disadvantages, were discussed in detail in Ref. [51]. Metals or metal oxides are easily processable via AM due to the development of technologies such as pulsed electric current sintering and PBF [52]. Therefore, the shape, porosity, and architecture of the monolith can be easily tuned for the specific catalytical process. Using a DMLS technology, a honeycomb monolith (carbon-based structures) from commercially available stainless steel (grade AISI 15-5PH) was used as support for broadly exploited Ni/CeO_2_–ZrO_2_ (Ni/cerium(IV) oxide–zirconium(IV) oxide) catalyst in the methane dry reforming process [53]. The catalyst revealed similar activity to industrially used cordierite catalyst. However, the main advantage was that printed metallic monolith did not require activation time, which follows from better heat transfer. Interestingly, the bare metallic monoliths exhibited significant activity at slightly increased temperatures, which was attributed to the intrinsic Ni content in AISI 15-5PH stainless steel. Similarly, a robust reactor printed from stainless steel via selective later melting technology was reported for a fast difluoromethylation reaction with *n*-butyllithium as a base and trifluoromethane as an atom-economic and inexpensive reagent. The use of stainless steel as the reactor body allowed the reaction to be carried out at −65 °C, resulting in a product with excellent yield > 80% with a total reaction time of less than 2 min [54].

**Figure 5:**
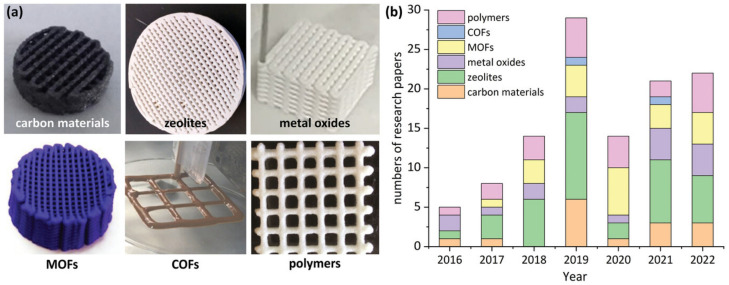
(**a**) Various materials applied in DIW fabrication for adsorption and catalytic applications: carbon materials, metal–organic frameworks (MOFs), polymers, covalent organic frameworks (COFs), zeolites, metal oxides. (**b**) The number of research papers published over the years, classified by material types. Reprinted with permission from Ref. [14]. Copyright [2023] Wiley.

The application of DIW in gas-phase adsorption and catalysis encompasses a wide range of material classes, including carbon-based materials, zeolites, metal oxides, metal–organic frameworks, covalent organic frameworks, and organic polymers (Figure 5). Among these, zeolites remain the most extensively investigated, whereas covalent organic framework-based systems are comparatively underexplored, highlighting a potential direction for future research.

Recent developments also extend toward the use of advanced functional materials, including hydrogels and biocomposites. These materials introduce additional functionalities such as high porosity, tunable chemical environments, and biological activity.

Metal–organic frameworks are nanoporous functional materials that have seen tremendous interest for catalysis, gas storage or separation, chemical sensing, electronic and ionic conduction, and biomedical applications [55]. Although metal–organic framework-based systems offer significant potential for catalysis and separation, their integration into AM remains at an early stage due to challenges associated with printability and mechanical stability.

The DIW process was used for UiO-66 Zr-based metal–organic framework composites, which were then thermally treated to obtain a mechanically stable but highly porous composite. The obtained monolith was tested for the catalytic decomposition of methyl paraoxon, imitating highly toxic organophosphate nerve agents [56]. A similar approach was used to construct 3D-printed hierarchical porous ceramics decorated with metal–organic frameworks nanoparticles. The catalytically active metal–organic frameworks nanoparticles MIL-100 (Fe) (MIL—Matérial Institute of Lavoisier) and HKUST-1 (Cu) (HKUST—Hong Kong University of Science and Technology) were in situ loaded on the framework to endow high catalytic performance. The obtained 3D-printed hierarchical porous ceramics loaded with MIL-100 and HKUST-1 were successfully used in the Fenton-like reaction for the catalytic degradation of various organic dyes such as rhodamine B, methylene blue, malachite green, and crystal violet [57,58]. The number of studies dedicated to printing metal–organic framework materials is high and it keeps on growing [59]. However, despite these few examples of 3D-printed metal–organic framework composites intended for organic synthesis applications, the use of AM of metal–organic framework-based materials still remains in the early stages.

DIW has also been reported as a feasible method for printing metal-based catalyst. For instance, a manganese–cerium–iron (Mn–Ce–Fe) monolith with hierarchical porosity, designed for the selective catalytic reduction in nitric oxide (NO) with ammonia at low temperatures, demonstrated superior performance [60]. DIW has also been applied to fabricate Ni/γ-Al_2_O_3_ monoliths for CO_2_ methanation. A study employing hydrogel-based inks enriched with Ni precursors showed that controlling parameters, such as Ni content and sintering temperature significantly affects catalyst decomposition, reduction rate, and overall performance [61]. The feasibility of the robocasting method for fabricating extremely robust 3D Fe/silicon carbide (SiC) monolithic catalysts for wet peroxide oxidation of phenol process using aqueous Si carbide-based ink followed by Fe-doping was also reported [62]. In order to develop a metal catalyst with the characteristics desired for their applications, post-modification approaches might also be combined. An example is the 3D-printed hierarchical nanoporous Cu prepared by a PBF technology followed by a dealloying process. The hierarchical nanoporous Cu architectures were fabricated by chemical dealloying the 3D-printed monolith from Cu–Mn alloy structures in ammonium sulfate ((NH_4_)_2_SO_4_). This versatile strategy enables the selective dissolution of Mn, leaving Cu atoms that spontaneously form a bicontinuous hierarchical porous structure through an intrinsic dynamic formation process. The obtained catalyst was tested as the electrocatalyst for methanol oxidation, where it displays highly efficient activity [63]. This approach was also studied in order to prepare catalysts for water purification. Using PBF technology 3D-printed catalyst device was created from Zr_55_Cu_30_Al_10_Ni_5_ alloy (bulk metallic glass) followed by a dealloying process to obtain a milli- and nano-scaled hierarchical porous structure. The resulting zirconium (Zr)-based catalyst demonstrated excellent catalytic properties towards the degradation of methyl orange in water [64].

A number of 3D-printed devices based on metal oxides or mixed metal oxides with metals have been reported for various catalytic applications. These include, in particular, the hydrogenation of alkynes and nitrobenzene (platinum(IV) oxide–tungsten(VI) oxide (PtO_2_–WO_3_)) [65], the photoproduction of hydrogen from a water–ethanol mixture in the gas-phase (gold/titanium(IV) oxide (Au/TiO_2_)) [66], the preferential oxidation of carbon monoxide (CO) (copper(II) oxide (CuO)/CeO_2_) [67,68], and the oxidation of methane (rhodium (Rh)/CeO_2_) [69]. Additional applications encompass azide–alkyne cycloaddition, the reduction of 4-nitrophenol to 4-aminophenol [70], Chan–Lam azidation (Cu/SiO_2_, Cu/silicon(IV) oxide (SiO_2_)), and cross-coupling reactions such as Sonogashir, Stille, and Suzuki (Pd/SiO_2_) [71,72], as well as ammonia decomposition (Ni/CeO_2_, Ni–Ru/CeO_2_) [73,74]. Furthermore, recent studies have explored the development of 3D-printed Ni–Cu sodalite catalysts for the sustainable production of γ-valerolactone from levulinic acid, with particular emphasis on the influence of Cu content and preparation methods on catalytic performance [75]. In a similar context, monolithic ceramic catalysts based on zirconium dioxide (ZrO_2_), fabricated via 3D printing, have been applied for the catalytic oxidation of volatile organic compounds, including aromatic mixtures such as benzene, toluene, ethylbenzene, and *o*-xylene [76].

To systematically evaluate the impact of AM on catalytic organic synthesis, the reported systems can be categorised based on (i) reaction type, (ii) catalyst composition, and (iii) fabrication strategy. These studies reveal clear structure–performance relationships, as summarised in Table 2.

From a technological perspective, different AM technologies offer distinct advantages depending on the target application. High-resolution methods such as DLP enable the fabrication of finely structured catalytic supports with controlled micro-scale porosity, whereas PBF and DMLS provide access to mechanically robust metallic reactors with superior thermal conductivity. The latter is particularly relevant for intensified processes, where efficient heat transfer is critical, such as methane dry reforming or highly exothermic hydrogenation reactions.

Another important trend is the increasing use of post-processing strategies, including impregnation, coating, and dealloying, to introduce catalytic functionality into printed structures. These approaches effectively decouple structural fabrication from catalytic activation, allowing greater flexibility in material design. For instance, hierarchical nanoporous metals obtained via dealloying of 3D-printed alloys exhibit high electrocatalytic activity, demonstrating that AM can be integrated with bottom-up nanostructuring techniques.

Despite these advances, several limitations remain. The majority of current AM-compatible materials exhibit limited chemical resistance, especially under harsh organic synthesis conditions. In addition, achieving an optimal balance between printability, mechanical stability, and catalytic performance remains a significant challenge. These constraints highlight the need for the development of new printable materials and hybrid fabrication strategies.

## 4. Customised Organic Devices

AM has enabled a paradigm shift in the design of devices for organic synthesis, moving from standardised glassware or metal toward customised, function-integrated systems. In contrast to traditional fabrication methods, AM allows the rapid prototyping of polymer-based flow devices with precisely defined geometries, enabling control over mixing, residence time, and multiphase interactions.

Polymeric and organic-based materials are increasingly employed not only as passive reactor components, but also as active elements in integrated chemical systems, where flow paths, valves, pumps, mixers, and separation units can be combined in a single device. The most straightforward approach is to design a flow path that enables the controlled transport and mixing of reactants (in liquid, gas or multiphase systems) in appropriate proportions for the reaction to occur [77,78,79,80]. A key advantage of polymer-based AM lies in its design flexibility and accessibility, enabling the incorporation of diverse functionalities within a single platform. However, this flexibility is counterbalanced by intrinsic limitations, particularly the relatively low thermal and chemical resistance of commonly used polymers, which remains a major constraint in their application to demanding organic synthesis conditions.

Metallic AM systems enable the direct fabrication of reactor–catalyst integrated devices, such as static mixers coated with active catalytic layers. Compared to conventional packed-bed systems, these structures offer improved mixing efficiency, reduced pressure drop, and enhanced scalability, making them particularly attractive for pharmaceutical and fine chemical production. Static mixers can be obtained via adaptative manufacture of metals to design catalysts. A series of 3D-printed metallic static mixer scaffolds, coated with an active catalyst layer with a wide range of catalytic applications were processed via the PBF technologies. The static mixers have been reported for various hydrogenation applications, such as the reduction in nitro groups [81,82], which is commonly used in manufacturing active pharmaceutical ingredients, hydrogenation of alkynes [83,84], reduction in fatty acids [85], and reductive aminations [86]. One representative example for utilising a 3D-printed static mixer is the use of commercially available SAE 316L-grade stainless steel as a catalyst scaffold, followed by deposition of a catalytically active Pd layer. The resulting system was demonstrated to be an efficient catalyst for the production of a key antimicrobial drug intermediate—linezolid (Zyvox^®^) [87].

One of the most significant advances enabled by AM is the development of integrated and automated synthesis platforms, in which reactionware fabrication and process execution are combined within a single workflow. Modified 3D printers have been repurposed as dual-function systems capable of both printing and reagent dosing, allowing the automated synthesis of pharmaceuticals such as ibuprofen or baclofen [88]. These studies demonstrate a broader trend toward the convergence of digital manufacturing and chemical synthesis, where reaction protocols can be translated directly into programmable fabrication instructions. Such an approach opens new opportunities for decentralised and on-demand production of fine chemicals. Newly developed robotic systems can be used to print, for example, polypropylene (PP) reaction vessels using the MEX technology. Ibuprofen synthesis can be performed at various scales by selecting the appropriate parameters in the robot’s control software. In the procedure, the reactants are preloaded into individual syringe pumps connected to the extruder of the 3D printer by the Teflon tubing. The syringe pumps are controlled by process-control software to feed the printer with reactants into the reactionware in the required volume and rate. A similar approach also deals with the synthesis of a muscle relaxant drug—baclofen [89]. The reaction system consisted of various connected 3D-printed devices that were designed to perform a particular role in the synthetic process (Micheal reaction, evaporation, extraction or filtration of intermediate products). These examples strongly demonstrate that AM might establish a solution that paves the way for the local production of drugs outside of specialist facilities.

The 3D printers commonly used for printing polymeric materials include MEX, PBF, and VPP technologies are capable of producing features smaller than 100 μm [10]. Therefore, AM can serve as a suitable base for micro- and milli-reactors. Polymer-based flow reactors represent the most widely explored class of AM-derived devices. Materials such as PP and ABS are commonly used due to their low cost and ease of processing, and have been successfully applied in a range of transformations, including photo-oxygenation, epoxidation, nucleophilic aromatic substitution, and imine synthesis [90]. The PP-based reactionware were reported in photo-oxygenation of 7-aminothienopyridinones [91], epoxidation of styrene [92], nucleophilic aromatic substitution (S_N_Ar) addition reactions, and the synthesis of bicyclic and tetracyclic heterocycles [93], and imines synthesis and reduction reactions with real-time analysis using attenuated total reflection infrared (ATR-IR) spectroscopy [94]. Nevertheless, a consistent limitation across these systems is their insufficient resistance to aggressive solvents and elevated temperatures. To address this challenge, high-performance polymers such as polyetheretherketone (PEEK) have been introduced, enabling the fabrication of reactors capable of operating under elevated pressure and temperature conditions. These systems allow access to intensified reaction regimes, including multistep flow synthesis and the use of superheated solvents, highlighting the importance of material selection in extending the applicability of AM in organic synthesis. Recently, in order to perform flow reactions at elevated temperatures, the milli- and micro-fluidic reactors made of PEEK were printed via MEX. The PEEK-based 3D-printed flow system was evaluated for a multistep reactions conversion of ribose derivatives to a 2-fluoro-arabinose derivative which is used as a precursor for the synthesis of nucleoside anticancer drugs. Further, the miniaturisation of printed channels, showed that it is possible to print reactors with channel dimensions under 500 µm [95]. The flow chemistry reactors with significantly enhanced thermal resistance and excellent mechanical strength for high pressure reactions have been produced using the AM process of MEX, from PEEK. Printed reactors have been shown to be suitable for flow chemistry and liquid–liquid extraction and are able to withstand pressures of at least 30 bar, allowing the use of superheated solvents [96].

**Figure 6:**
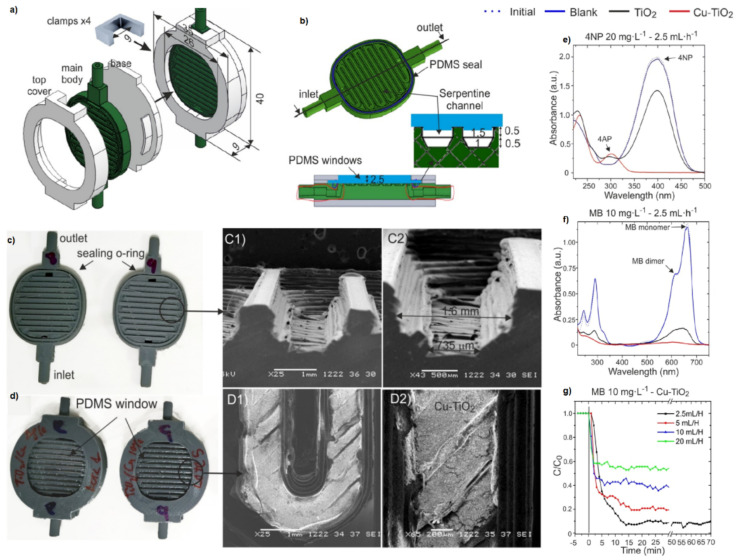
(**a**) Assembly scheme of the microreactor parts including clamps for holding the group together and (**b**) main body, top view, and cross-section of the serpentine channel. Photographs of 3D-printed devices: (**c**) main bodies and (**d**) assembled microreactors. SEM images of the serpentine channel: (C1 and C2) cross-section with trapezoidal shape prior to photocatalyst nanoparticle coating and (D1–D2) top view of a channel coated with the catalyst. Catalytic activity of the microreactor for: (**e**) photoreduction of 20 mg·L^−1^ 4-nitrophenol (4NP) to 4-aminophenol (4AP), (**f**) photodegradation of 10 mg·L^−1^ methylene blue (MB), (**g**) photodegradation of 10 mg·L^−1^ MB at different feed flow rates (2.5, 5, 10, and 20 mL·h^−1^). Reprinted with permission from Ref. [97]. Copyright [2020] American Chemical Society.

Beyond passive flow devices, AM has enabled the development of functionalised microreactors in which catalytic activity is incorporated directly into the reactor architecture. The ability to fabricate complex channel geometries, such as serpentine or lattice structures, significantly enhances mixing efficiency, mass transfer, and, in the case of photocatalytic systems, light distribution. As a result, reactor geometry emerges as a critical parameter governing process performance, often comparable in importance to catalyst composition. This is exemplified by polymer-based microreactors incorporating TiO_2_-based photocatalysts, which demonstrate efficient degradation of organic pollutants due to improved light harvesting and flow characteristics. The ABS-based flow reactors were established as remarkably stable for catalytic purposes. Moreover, relatively low cost commonly available ABS filament and the short time of AM promote design creativity. For instance, a printed microfluidic device has been demonstrated as an effective tool for addressing one of the major 21st-century environmental challenges—water contamination. Interestingly, a low-cost, fast and easy-to-build microreactor was reported for photocatalytic applications based on three modules. The first was a main body printed directly from ABS with a serpentine pattern of microchannels, in which the TiO_2_ or Cu–TiO_2_ nanoparticles photocatalyst was placed, and a top holder with a transparent polymer window and a part-mounting base (Figure 6) [97]. The microfluidic device demonstrated the photocatalytic degradation of two common water pollutants (methylene blue and 4-nitrophenol) with excellent performance.

Since printing or modifying an object only requires changing the computer software file, the technology can be easily transferred, enabling the widespread use of such sophisticated printed devices worldwide as needed. A number of the flow chemistry devices constituted from ABS have been reported to date, including biodiesel synthesis [98], continuous acid-catalysed esterification for reducing free fatty acid content in mixed crude palm oil [99], photocatalytic degradation of organic pollutants (rhodamine 6G) [100], and micro free-flow electrophoresis for separations of fluorescent dyes (myoglobin and cytochrome c) [101]. The miniaturisation of technologies has driven the need for high precision techniques in microdevice manufacture, where 3D printing has emerged as a powerful tool. By enabling the creation of photocatalysts and supports with complex geometries, 3D printing has revolutionised the field of photocatalysis, allowing for the design of reactors and catalysts that optimise light harvesting efficiency, chemical conversion, and flow regimes [9].

Besides vinyl-based copolymers, (bio)degradable polymers and organic-based materials have been employed for flow chemistry devices. These include PLA flow reactors for enantioselective catalytic synthesis of pharmaceutically active chiral 1,2-amino alcohols [102], glycosylation reactions [103], elimination of the propargyloxy protecting group from proc-4-methoxybenzylamine [104], printable chitosan hydrogel for photodegradation of phenol, and emerging persistent water micropollutants [105] or 3D-printed temperature-responsive poly(*N*-iso-propylacrylamide) reactors for exothermic emulsion copolymerisation of vinyl acetate and a vinyl neodecanoate [106].

**Figure 7:**
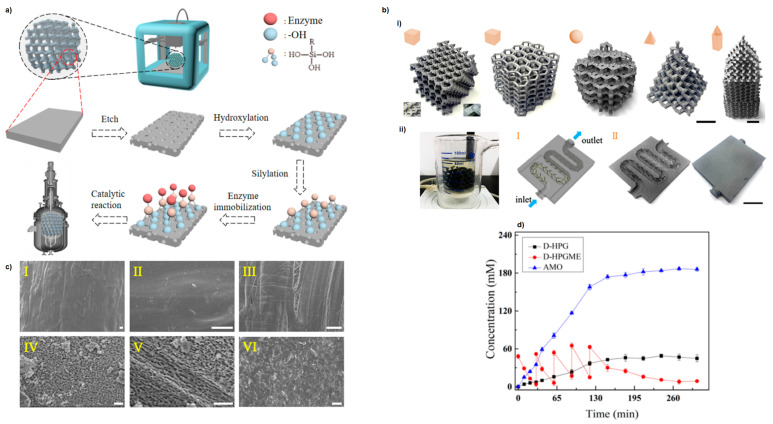
Schematic diagram of enzyme immobilisation on chemically modified 3D-printed scaffolds. (**a**) 3D-printed carbon fibre reinforced PLA (C-PLA) was etched with piranha solution and hydroxylated with peracetic acid, followed by reacting the surface hydroxyl groups with a silane coupling agent to generate covalent linking points or adsorption sites for the enzyme. After enzyme immobilisation, the 3D-printed scaffolds were assembled into enzymatic reactors. (**b**) 3D-printed scaffolds for enzyme immobilisation. (i) Digital images of 3D-printed scaffolds. (ii) An integrated reactor with an enzyme-immobilised 3D-printed carrier for biocatalysis. (I) All 3D-printed microfluidic reactors with scaffolds in the channel. Schematic representation of a 3D-printed reactor designed in 3D Max 2014. (II) Digital images of the 3D-printed reactor (profile structure (left) and appearance (right)). Scale bar: 2 cm. (**c**) SEM images of C-PLA: (I, II) original C-PLA (I, low magnification; II, high magnification); (III) C-PLA hydroxylated with peracetic acid; (IV) C-PLA etched with piranha solution; (V) C-PLA etched with piranha solution and then hydroxylated by peracetic acid; (VI) C-PLA with immobilised enzyme after etching and hydroxylation. Scale bars are 200 μm in (I–III) and 20 μm in (IV–VI). (**d**) Time course of amoxicillin (AMO) synthesis by immobilised enzymes. Reaction conditions: 200 mM 6-aminopenicillanic acid (6-APA) in 50 mM buffer at 20 °C (pH 6.5) and *D*-hydroxyphenylglycine methyl ester (D-HPGME) (240 mM). Adapted with permission from Ref. [109]. Copyright [2019] American Chemical Society.

The tunability of AM makes it possible to design catalysts tailored for specific biotechnological processes. A prominent example is the direct 3D printing of tuneable living inks for alcoholic fermentation. The bioink was prepared from nanocellulose and freeze-dried baker’s yeast, which are capable of producing ethanol via glucose fermentation. Using DIW technology, well-defined macroporous structures (e.g., periodic lattices and radial arrays) were fabricated from the bioink. These structures remained metabolically active for up to 4 months and exhibited local proliferation, enabling more efficient bioprocessing compared to bulk counterparts due to improved mass transfer through the designed porous architecture. The ability to print living cells represents a significant advancement for industrial biotechnology applications, with implications for the production of pharmaceuticals, food, and biofuels [107]. Hydrogel-based systems have further enabled the fabrication of biocatalytic reactors, in which enzymes or living cells are immobilised within 3D-printed scaffolds. Such systems demonstrate enhanced process efficiency due to improved mass transfer and stability, highlighting a promising direction for the integration of chemical and biological catalysis. In this context, the immobilisation of enzymes onto porous 3D-printed scaffolds is an emerging area of research. The use of polyester-based scaffolds as supports for catalytically active enzymes has also been reported. The model enzymes glucose oxidase and peroxidase were immobilised in macroporous poly(ε-caprolactone) (PCL) monolith to produce a simple, low-cost, flexible reactor for continuous-flow bioprocesses [108]. The carbon fibre-reinforced PLA scaffolds with a high specific surface area were also established as an excellent host for various enzymes (protease, lipase, penicillin G acylase or glycosidase) [109]. 3D-printed PLA scaffolds were assembled in enzymatic reactors. PLA was first etched with a piranha solution and hydroxylated with peracetic acid, and then the hydroxyl groups from surface were reacted with a silane coupling agent to generate covalent binding or enzyme adsorption sites. The obtained devices were tested in enzyme-dependent bioprocesses (amoxicillin or lactosucrose synthesis), which showed striking activity and stability compared to that of their native counterparts in the solution (Figure 7).

Besides polyesters, other biomaterials have been employed for the fabrication of 3D-printed scaffolds as hosts for enzymes, including sodium alginate hydrogel for immobilisation of laccase [110], glucose oxidase and catalase [111], xylanase [112], aldo-keto reductase [113], agarose-based modules containing an esterase or an alcohol dehydrogenase [114], phenacrylate decarboxylase [115], polyHIPEs (HIPEs—porous emulsion-templated polymers synthesised within high internal phase emulsions) for β-*D*-galactopyranoside immobilisation [116], copolymer of poly(acrylic acid) and poly(ethylene glycol) (PEG) diacrylate hydrogel as host for β-galactosidase [117], and PEG diacrylate-based hydrogel system for β-galactosidase entrapment [118].

The diversity of AM does not limit one’s creativity. An interesting emerging concept is the fabrication of catalytically active tools, such as functionalised stirrers and modular inserts, which combine mechanical and catalytic functions within a single device. These unconventional systems highlight the versatility of AM and its potential to redefine reactor design beyond traditional configurations. While these approaches are still largely at the proof-of-concept stage, they demonstrate the ability of AM to enable novel reactor concepts that are not accessible using conventional manufacturing technologies. The series of stirrers with catalytically active pieces were demonstrated for various reactions; in particular, 3D-printed sleeves for magnetic stirring bars: from PP base mixed with Pd catalyst for Suzuki–Miyaura cross-coupling reactions [119], high surface area holder for a magnetic stirrer bead containing *p*-toluenesulfonic acid for catalysing Mannich reactions [120], stir bar sleeves from PP framework mixed with catalytically active powdery Pd/SiO_2_ for hydrogenation reactions [121], catalytically active structures from acrylic acid resins containing carboxylic acid, amine, and Cu carboxylate functional groups for catalysing the Mannich, aldol condensation or Huisgen cycloaddition reactions [122], and catalytically active devices from commercially available methacrylate-based resin loaded with Schreiner’s thiourea for organocatalysed Friedel–Crafts alkylation [123].

Overall, the application of AM in customised organic devices demonstrates a clear transition from passive reactor fabrication toward integrated, multifunctional chemical systems. While polymer-based platforms dominate due to their accessibility, their broader implementation remains limited by material constraints. Future progress will depend on the development of chemically resistant printable materials, the integration of catalytic and sensing functionalities, and the advancement of digitally controlled, autonomous synthesis platforms. These developments will be essential for transforming AM into a key enabling technology for sustainable and distributed chemical manufacturing.

## 5. Future of Additive Manufacturing in the Field of Organic Synthesis—Final Conclusions

The rapid development of AM has significantly expanded its application across organic chemistry, enabling the rapid and cost-effective fabrication of customised laboratory devices, microfluidic systems, electrochemical reactors, and digitally controlled synthesis platforms. By allowing precise control over geometry, material distribution, and process parameters, AM has introduced new possibilities for reaction engineering, catalyst design, and the development of advanced functional materials. In particular, the integration of AM with flow chemistry and electrosynthesis has opened new pathways for sustainable transformations, including the valorisation of biomass-derived feedstocks into value-added chemicals and fuels [4,124].

In catalysis, AM has enabled the fabrication of novel structures and materials that are difficult or impossible to achieve using conventional manufacturing methods. The ability to produce graphene-based materials, monoliths, and metal–organic frameworks with controlled architectures offers significant potential for improving catalytic performance. Moreover, AM facilitates not only the synthesis of organic compounds but also the production of low-cost, tailor-made reactors and laboratory devices adapted to specific chemical processes [125]. Overall, its versatility, cost-effectiveness, and design flexibility make AM a powerful tool for addressing the evolving needs of modern chemical research.

Despite these advances, the implementation of AM in organic synthesis remains associated with several critical limitations. One of the most significant challenges is the limited chemical and thermal resistance of commonly used printable materials, particularly polymers, which restricts their use under harsh reaction conditions. Although high-performance materials such as PEEK or ceramic-based systems offer improved stability, their processing remains more complex and less accessible. In addition, material compatibility with reactive intermediates, solvents, and catalysts continues to be a key constraint, often requiring extensive post-processing or surface modification.

Another important limitation lies in the trade-off between resolution, scalability, and material properties across different AM technologies. While methods such as SL and DLP provide high precision, particularly in microfluidic applications, they are often limited by the chemical stability of photopolymer resins. In contrast, technologies such as MEX offer accessibility and low cost but suffer from lower resolution and poorer surface quality. Powder-based methods (e.g., SLS, PBF) provide improved mechanical strength and chemical resistance; however, they are associated with higher energy consumption and equipment costs. Consequently, no single AM technology currently satisfies all requirements for organic synthesis, highlighting the need for hybrid and multi-material manufacturing approaches.

From a process perspective, challenges also remain in the standardisation, reproducibility, and scalability of AM-fabricated systems. Variations in printing parameters, material batches, and post-processing steps can significantly affect device performance, limiting their translation from laboratory-scale demonstrations to industrial applications. Furthermore, while AM enables the fabrication of complex reactor geometries, the relationship between structure and performance is not yet fully understood, particularly in multiphase and catalytic systems.

From a sustainability perspective, the choice of AM technology also plays a significant role. MEX technologies generate relatively low material waste and enable the use of biodegradable polymers or hydrolytically degradable polymers such as PLA, although their limited durability may reduce long-term sustainability. In contrast, powder-based technologies allow partial material reuse but are associated with higher energy consumption. Therefore, future advancements in AM for organic chemistry should focus not only on improving chemical performance but also on minimising environmental impact.

Overall, the application of AM in organic synthesis demonstrates a clear shift toward process intensification and functional integration. The most significant advances are observed when structural design (e.g., channel geometry, porosity) is intentionally coupled with catalytic functionality. Future key research directions in this field should focus on the development of chemically robust, multifunctional, and printable materials, which will be essential for expanding the applicability of AM in organic synthesis. Furthermore, the integration of catalytic, sensing, and control functionalities within a single device will enable the next generation of smart reactors and autonomous synthesis platforms. Also, advances in digitalisation and process automation, including the coupling of AM with artificial intelligence and real-time monitoring, may facilitate fully programmable and decentralised chemical manufacturing. Finally, a deeper understanding of structure–performance relationships in 3D-printed systems will be crucial for the rational design of efficient and scalable reactors.

In summary, AM is transitioning from a prototyping tool to a transformative technology in organic synthesis, enabling process intensification, functional integration, and design flexibility. However, overcoming current material, technological, and scalability limitations will be essential to fully realise its potential in sustainable chemical process design and industrial implementation.

## Figures and Tables

**Table 1:** Comparison of AM technologies used in organic chemistry [1,2,11,12,13,14,15,16,17,18,19,20,21,22,23,24,25].

AM Technology	Working Principle	Typical Materials	Resolution ^1^	Chemical Resistance	Advantages	Limitations	Representative Applications in Organic Chemistry
Material extrusion	Extrusion of molten thermoplastic through a heated nozzle	PLA, ABS, PETG, polypropylene (PP)	Low-moderate (~100–300 µm)	Low-moderate (limited solvent resistance)	Low cost, widely available, rapid prototyping	Low resolution, anisotropy, poor chemical resistance	Reaction vessels, simple flow reactors, lab accessories
Vat photopolymerisation (SL/DLP)	Photopolymerisation of liquid resin using UV light	Epoxy/acrylate-based resins	High (~25–100 µm)	Low (often sensitive to organic solvents)	High precision, smooth surfaces, complex geometries	Limited chemical resistance, post-processing	Microreactors, microfluidics, precise flow systems
Powder bed fusion (SLS)	Laser sintering of polymer powder layers	Polyamides, thermoplastic polyurethanes, composites	Moderate (~80–150 µm)	Moderate–high	No supports, good mechanical strength, complex structures	High cost, limited availability of materials	Durable reactors, catalytic supports, structural components
Powder bed fusion (DMLS)	Laser sintering of metal powders layer-by-layer	Stainless steel, Ti alloys, Al alloys	Moderate (~50–150 µm)	High	Excellent mechanical strength, high thermal conductivity, suitable for harsh conditions	High cost, surface roughness, post-processing required	Metallic reactors, catalyst supports, high-temperature flow systems
Material jetting/inkjet printing	Deposition of droplets of photopolymers, cured by UV	Photopolymers (multi-material)	Very high (~20–50 µm)	Low–moderate	Multi-material printing, high accuracy	Expensive equipment, limited chemical resistance	Lab-on-a-chip, integrated reactor systems
Direct ink writing	Extrusion of viscoelastic inks (often functional)	Ceramics, hydrogels, catalytic inks	Moderate (~50–200 µm)	High (depending on the composition)	Possibility of printing functional materials (e.g., catalysts)	Requires rheology optimisation, lower precision	Catalytic structures, functional reactors

^1^ Resolution values are approximate and depend on printer specifications and process parameters.

**Table 2:** Summary of organic synthesis processes and applicable monolithic catalysts.

Reaction/Process	Catalyst System	AM Technology	Structural Feature	Key Advantage	Refs.
Methanol to olefin	Metal-doped ZSM-5 monoliths, SAPO-34 zeolite	Robocasting/DIW	Hierarchical porosity	Enhanced activity and selectivity	[27,28,29,30]
*n*-Hexane cracking	Zeolite monoliths	3D printing	Structured channels	Improved selectivity vs. powder catalysts	[31]
Methanol to dimethyl ether	Zeolite-based catalysts	3D printing	Controlled acidity	Improved catalytic performance	[32,33]
CO_2_ to dimethyl ether	CuO–ZnO–ZrO_2_/zeolite	Robocasting	Tailored porous structure	Efficient CO_2_ utilisation	[34]
Oxidative propane dehydrogenation	Mixed oxide/zeolite systems	3D printing	Tunable composition	High conversion efficiency	[35,36]
Toluene combustion	Co_3_O_4_ on monolith	DLP	Micro-scale porosity	High catalytic activity	[37]
α-Pinene isomerisation	Zeolite-coated monoliths	DLP	Tunable Si/Al ratio	Controlled selectivity	[38]
Catalytic cracking (fuels)	Zeolite-functionalised metal lattice	PBF	3D lattice	High thermal stability	[39]
Lewis acid-catalysed reactions	Al_2_O_3_ (Al-based Lewis acids)	3D printing	High surface area porous structure	Versatile catalytic support and intrinsic Lewis acidity	[40]
Biginelli/Hantzsch reactions	Al_2_O_3_ monolith	Robocasting	Woodpile structure	Increased yield (~20%)	[41]
Methanol reforming (H_2_ production)	γ-Al_2_O_3_	DLP	Ordered channels	Efficient hydrogen production	[42]
Hydrogenation (e.g., isopulegol to menthol)	Metal/Al_2_O_3_ (Pd, Pt, Ni, etc.)	3D printing	Flow reactor design	High yield and selectivity	[43]
Ullmann coupling	Cu/Al_2_O_3_	3D printing	Porous monolith	Enhanced reactivity	[44]
Cross-coupling reactions	Pd/Al_2_O_3_	3D printing	Structured catalyst	Improved catalytic efficiency	[45]
Oxidative coupling of methane	Mn–Na_2_WO_4_/Al_2_O_3_	3D printing	Structured porous monolith	Improved selectivity and thermal stability	[46]
CO_2_ methanation	Ni/Al_2_O_3_, Cu/Al_2_O_3_	3D printing	Structured support	Improved conversion	[47,49]
Oxidation of benzyl alcohol to benzaldehyde	Fe–Co alloy, Fe–Co/Al_2_O_3_, Fe–Pd/Al_2_O_3_	3D printing	Structured porous support	Enhanced catalytic activity and selectivity	[50]
Methane dry reforming	Ni/CeO_2_–ZrO_2_ on steel	DMLS	Honeycomb monolith	Improved heat transfer	[53]
Difluoromethylation	Stainless steel reactor	SLM	Integrated reactor	Fast reaction (<2 min)	[54]
NO reduction	Mn–Ce–Fe monolith	DIW	Hierarchical porosity	Low temperature process	[60]
Phenol oxidation	Fe/SiC monolith	Robocasting	Robust structure	Efficient oxidation	[62]
Methanol oxidation (electrocatalysis)	Nanoporous Cu (dealloyed)	PBF + dealloying	Hierarchical porosity	High catalytic activity	[63]
Water purification	Zr-based porous catalyst	PBF + dealloying	Multi-scale porosity	Efficient pollutant degradation	[64]
CO oxidation	CuO/CeO_2_	3D printing	Mixed oxide	High catalytic activity	[67,68]
Methane oxidation	Rh/CeO_2_	3D printing	Supported catalyst	High efficiency	[69]

## Data Availability

No new data were created or analyzed in this study. Data sharing is not applicable to this article.
